# A Positive Emotion–Focused Intervention to Increase Physical Activity After Bariatric Surgery: Protocol for a Pilot Randomized Controlled Trial

**DOI:** 10.2196/39856

**Published:** 2022-10-06

**Authors:** Emily H Feig, Lauren E Harnedy, Anne N Thorndike, Christina Psaros, Brian C Healy, Jeff C Huffman

**Affiliations:** 1 Department of Psychiatry Massachusetts General Hospital Boston, MA United States; 2 Harvard Medical School Boston, MA United States; 3 Department of General Internal Medicine Massachusetts General Hospital Boston, MA United States; 4 Department of Neurology Brigham and Women's Hospital Boston, MA United States

**Keywords:** behavioral intervention, physical activity, positive psychology, design, bariatric surgery, positive, psychological, well-being, weight loss, intervention, feasibility, acceptability, efficacy, effect, obesity, weight

## Abstract

**Background:**

Physical activity levels after bariatric surgery are usually low, despite the significant protective health benefits of physical activity in this population. Positive psychological well-being is associated with improved adherence to health behaviors, but bariatric surgery patients often have negative associations with physical activity that prevent sustained engagement.

**Objective:**

The Gaining Optimism After weight Loss Surgery (GOALS) pilot randomized controlled trial is aimed at testing a novel intervention to increase physical activity after bariatric surgery, which incorporates positive psychological skill-building with motivational interviewing and goal-setting.

**Methods:**

The GOALS trial is a 2-arm, 24-week pilot randomized controlled trial that aims to enroll 58 adults who report less than 200 minutes per week of moderate to vigorous physical activity and a desire to become more active 6-12 months after bariatric surgery. GOALS is testing the feasibility, acceptability, and preliminary efficacy of a positive psychology–motivational interviewing telephone intervention targeting to increase physical activity and associated positive affect. Intervention components include positive psychology, goal-setting, self-monitoring via provided Fitbits, and motivational interviewing to overcome barriers and increase motivation. The intervention is compared to a physical activity education control that includes mailings with psychoeducation around physical activity and provision of a Fitbit. The primary outcomes of the pilot trial are feasibility and acceptability, measured as session completion rates and participant ratings of ease and helpfulness of each session. The main secondary outcome is change in accelerometer-measured moderate to vigorous physical activity post intervention and at 24-week follow-up. Additional outcomes include changes in attitudes related to physical activity, psychological well-being, and physical health measures.

**Results:**

This multiphase project was funded in 2020 and institutional review board approval was obtained for the proposed trial in 2021. Recruitment for the randomized controlled trial began in July 2022. Upon completion of the pilot trial, we will examine the feasibility, acceptability, and preliminary efficacy of the intervention.

**Conclusions:**

Although bariatric surgery is the most effective treatment available for severe obesity, weight regain occurs, often in the context of low psychological well-being. Many individuals would benefit from learning strategies to increase positive psychological well-being after bariatric surgery, which could help them maintain lifestyle changes. Positive psychology is a novel approach to improve adherence by increasing positive associations with health behaviors including physical activity. The GOALS pilot trial will determine whether this type of intervention is feasible and acceptable to this population and will provide a foundation for a future full-scale randomized controlled efficacy trial.

**Trial Registration:**

ClinicalTrials.gov NCT04868032; https://clinicaltrials.gov/ct2/show/NCT04868032

**International Registered Report Identifier (IRRID):**

PRR1-10.2196/39856

## Introduction

Bariatric surgery is the most effective treatment available for severe obesity, often resulting in cost-effective, sustained weight loss [1-3]. However, approximately 25% of surgical patients do not achieve long-term weight loss maintenance [2-4]. Weight loss is associated with remission of weight-related comorbidities (eg, type 2 diabetes, hypertension, and hypercholesterolemia), and weight loss maintenance is vital for preserving these improvements in health [3]. Physical activity is critical for weight loss maintenance and improved health after bariatric surgery, particularly given the increased risk of cardiometabolic disease in this population [5-7].

After bariatric surgery, experts recommend individuals to engage in at least 150 minutes per week of moderate to vigorous physical activity (MVPA), and even higher levels may be needed to control weight [8-10]. Unfortunately, a large majority of people who have bariatric surgery do not meet this recommendation [11-13]. Increasing physical activity, even without weight change, can improve insulin sensitivity, cardiorespiratory fitness, blood pressure, and blood lipid levels, all of which confer a lower risk for cardiac and metabolic disease in the general population [14-17]. Behavioral interventions to improve physical activity after bariatric surgery show promise, but the evidence is still limited by a lack of trials that do not include an in-person exercise program and are well-powered with long-term follow-up [18-20].

Emotional factors play a role in physical activity engagement and health outcomes. Psychological distress predicts lower physical activity levels and less weight loss after bariatric surgery [21], and these individuals are more likely to experience depressive symptoms than the general population [22,23]. Conversely, positive psychological constructs such as optimism and positive affect are associated with improved health independent of depression but have not been examined thoroughly in people who have bariatric surgery [24-26]. Further, positive affect during physical activity has been shown to predict future physical activity, supporting the “upward spiral” theory of lifestyle change [27,28]. This theory posits that by experiencing positive affect when performing a health behavior, nonconscious motives increase one’s likelihood of repeating that behavior. Over time, health behaviors become reinforcing rather than burdensome. However, most people experience a decrease in positive affect during exercise [29], and sedentary women with obesity have been found to experience even lower pleasure during physical activity than those with a BMI in the normal or overweight category [30]. People undergoing bariatric surgery may be missing out on this “upward spiral” owing to emotional barriers to physical activity, such as anxiety about getting injured, shame about appearance, and experiences of weight stigma, as well as physical barriers such as increased shortness of breath and discomfort [31-33]. This group would benefit from new skills to increase positive affect during physical activity as well increased psychological well-being in general.

Physical activity interventions that include positive psychology may be particularly effective after bariatric surgery. In addition to improving overall well-being, positive psychology interventions could improve physical activity by targeting positive affect during physical activity engagement [34,35]. Positive psychology may be more effective in combination with an adherence-based program such as motivational interviewing, a technique that focuses on clarifying motivation, addressing ambivalence, and setting achievable goals [36]. Indeed, a combined, remotely delivered positive psychology–motivational interviewing (PP-MI) intervention has shown preliminary efficacy in improving health behaviors in patients with type 2 diabetes and those with heart disease [37-39]. However, this intervention does not address the unique barriers that are common after bariatric surgery (eg, history of negative experiences with exercise due to injuries or weight stigma, adapting to a drastically changing body, and managing excess skin after weight loss) and does not directly address affect during physical activity.

The Gaining Optimism After weight Loss Surgery (GOALS) pilot randomized controlled trial (RCT) is testing an adapted PP-MI intervention promoting physical activity in individuals who have undergone bariatric surgery in the past 6-12 months. This paper describes the design and development of the intervention.

## Methods

### Overview

The GOALS trial is a 2-arm, 24-week pilot RCT that tests the feasibility, acceptability, and preliminary efficacy of a PP-MI telephone intervention, in comparison to a control arm, on physical activity and psychological, behavioral, and physical health outcomes immediately post intervention and at 24-week follow-up. The primary outcome of the trial is feasibility and acceptability of the intervention as measured by session completion rates and participant ease and utility ratings. The secondary outcomes are change at postintervention 10-14 weeks and 24-week follow-up in MVPA measured using an accelerometer. Additional outcomes include changes in light physical activity and steps per day, attitudes related to physical activity (enjoyment, self-efficacy, perceived barriers, and exercise identity), psychological well-being (symptoms of depression and anxiety, optimism, positive affect, internalized weight bias, and general self-efficacy), and health measures (exercise capacity, BMI, bariatric surgery behavioral adherence, and general health status) post intervention and at 24-week follow-up.

### Ethics Approval

The institutional review board of the Mass General Brigham initially approved the multiphase study in 2020 (2021P001006).

### Study Development

The GOALS intervention and study protocol were developed and refined in accordance with the Obesity-Related Behavioral Intervention Trials (ORBIT) model [40]. The ORBIT model focuses specifically on early, pre-efficacy phases of intervention development and emphasizes a flexible, iterative approach to moving between phases. The GOALS intervention is based on a PP-MI intervention that was initially developed for people with heart disease [37]. To adapt the intervention for the unique experiences of those who have bariatric surgery, a qualitative study was performed to understand the emotional experiences of people with a recent history of bariatric surgery regarding physical activity (ORBIT phase 1a and 1b; design: define and refine) [33]. Results from this study informed adaptation from the original PP-MI intervention to develop the GOALS intervention. Next, a proof-of-concept trial of the newly developed intervention was completed in 12 participants with exit interviews to refine study procedures and intervention content (ORBIT phase IIa; preliminary testing: proof of concept). Results from this study phase led to further adjustments in the intervention that is now being tested in the described pilot RCT (ORBIT phase IIb; preliminary testing: pilots). These include content changes to address additional common barriers to physical activity (eg, history of injuries), adjusting the positive psychology content to include some general exercises in addition to those focused on physical activity, and some small changes to session order and organization.

### Population

The GOALS trial is enrolling adults (age 18+ years) with a history of bariatric surgery in the prior 6-12 months. They also must self-report less than 200 minutes per week of MVPA and a desire to increase physical activity. While the physical activity recommendation is 150 minutes per week of MVPA, we chose a higher cutoff owing to a high likelihood of overestimation in self-reported physical activity [12], along with additional benefits of higher activity levels for weight loss promotion and maintenance [9,10]. Participants must have telephone access for study sessions and be able to read and speak English. Individuals are excluded from the study if they have cognitive deficits that preclude participation or informed consent assessed using a 6-item assessment tool designed to assess suitability for research participation [41], illness likely to lead to death in the next 6 months per chart review, inability to be physically active (eg, severe arthritis), severe psychopathology that may limit the ability to participate in the study per chart review, or current participation in another program targeting physical activity besides the standard care they receive at their surgery center.

### Participant Recruitment

Our goal is to randomize 58 participants. We identify patients with surgery dates in the relevant time frame using the hospital system’s Research Patient Data Registry, which allows for searching of electronic medical records to extract lists of patients who meet certain criteria. We send opt-out letters to patients from this list by mail and through the patient portal. Letters briefly describe the study and provide contact information if patients want to decline further contact. Letters are followed by a recruitment phone call 2 weeks later for those who do not opt out. We also can advertise for the study using a flyer to be distributed during postoperative groups and visits within the bariatric surgery clinic. These recruitment methods have been used successfully in prior studies [33].

### Participant Screening

Interested participants complete a screening phone call that includes a version of the International Physical Activity Questionnaire–Short Form modified to include brisk walking as a form of moderate activity [42], a 6-item cognitive deficit assessment [41], and questions about interest and ability to increase physical activity and participation in any other physical activity program. If eligible, their baseline visit is scheduled at this time.

### Assessment Visits

Participants attend assessment visits at baseline, end of treatment (10-14 weeks), and follow-up (24 weeks) at the hospital’s translational clinical research center. Assessment visit timing is designed to assess both the immediate and sustained intervention impact. Informed consent is obtained at the baseline visit. At each assessment, participants provide demographic and medical information (eg, medical comorbidities and weight history) and complete self-report measures via REDCap. Physiological measures are obtained by a trained translational clinical research center staff member, and 5-mL samples of blood are drawn. Staff also perform a 6-minute walk test to assess functional exercise capacity [43]. Participants are asked to wear an ActiGraph GT3X-BT accelerometer [44] for 7 days (minimum acceptable use is 4 days with 10 hours of recorded data) at each assessment. Participants are paid US $100 for completing each assessment visit.

### Randomization Visit

After wearing the accelerometer for 7 days following the baseline visit, participants return the accelerometer and are randomized 1:1 with a random number generator to a study condition (PP-MI or control) after sufficient wear time is confirmed. Only participants who complete accelerometry and return for this visit are randomized. At this time, study staff provide the participant a Fitbit to aid with self-monitoring physical activity and helps them set it up with a study-created account. If they are randomized to PP-MI, they are given the study manual and meet with a study interventionist for approximately 45 minutes for an in-person discussion of the introduction and first session of the program, including setting a long-term physical activity goal to reach by the end of the program. If they are randomized to the control group, they are provided an educational handout about physical activity.

### Intervention Components

The GOALS intervention was adapted and refined on the basis of results from the formative qualitative study and from interventionist experience and participant feedback from a proof-of-concept trial of the intervention [33]. It is delivered over 10 weeks via weekly 30-45–minute phone calls supported by a written participant manual. A window of 14 weeks for intervention completion allows for flexibility in the timing of weekly sessions. Each week includes a topic related to increasing physical activity and a positive psychology skill that is focused on increasing positive emotions in general and during physical activity (see Table 1). Participants are assigned pages in the manual to read and worksheets to complete each week. The physical activity portion of the call includes a review of the prior week’s physical activity topic and of their activity levels from the prior week, including whether they met their goal based on Fitbit data or other methods of self-monitoring, and noting positive emotions experienced during physical activity that week. Furthermore, a new topic is assigned to be completed over the subsequent week and a new physical activity goal is collaboratively set for the upcoming week on the basis of their activity level in the prior week. This goal is customized to each participant’s current activity level and interest, and is primarily self-determined with input from the interventionist as needed. The positive psychology portion includes a review of written assignments from the prior week and associated positive thoughts and feelings identified, followed by introduction to the next week’s topic and assignment. Reviewing, reflecting, and planning for the future sessions encourage integration of positive psychology skills learned into daily life by developing a specific plan to build a habit. All content is delivered using a motivational interviewing approach. The specific weekly topics are described in Table 1. Participants are also sent psychoeducation about physical activity via mail or email as in the control condition (see the *Control Content* section for details).

**Table 1:** Weekly intervention topics.

Week	Physical activity topic	Positive psychology topic
1	*Getting started with increasing activity*: benefits of physical activity, importance and confidence in making a change, and setting an overall program goal	*Identifying positive feelings during exercise*:^a^ pay attention to and write down specific positive emotions during and after physical activity
2	*Pros and cons of change/SMART*^b^*goals*: consider pros and cons of making a behavior change and of staying the same, setting goals that are specific, measurable, attainable, relevant, and time-based	*Gratitude for positive events*: identify 3 good things that happen this week, one related to exercise and two broadly, write about them and associated positive thoughts and feelings
3	*Barriers and problem-solving*: identify barriers to physical activity and brainstorm ways to overcome them	*Positive reappraisal – general*: learn about positive reappraisal as a way of seeing the silver lining in a negative situation, use it in response to a situation this week following guided prompts
4	*Strength training and equipment resources*: set a strength-training goal in addition to general physical activity goal; identify and use exercise equipment	*Positive reappraisal for physical activity*: consider common negative experiences with physical activity and how to positively reappraise; use positive reappraisal for one situation related to physical activity this week
5	*Neighborhood, online, and social resources*: brainstorm resources for increasing activity and use a new one this week	*Using perseverance*: review benefits of perseverance, pick physical activity-related goal to achieve using perseverance this week
6	*Reviewing and reflecting*: review progress so far, adjust long-term goals if needed, and reassess importance and confidence	*Reviewing and reflecting*: review skills learned so far, make a plan to integrate one into daily life this week to build a habit
7	*Reducing sedentary time*: assess most sedentary activities and make a plan to incorporate standing breaks and small increases in movement throughout the day	*Focusing on meaning during physical activity*: identify nonweight reasons for physical activity and practice thinking of these motivators when making the decision to be active and during activity this week
8	*Managing slips*: normalize slips, plan how to avoid long-term decreases after slips	*Remembering past successes*: write about a time in the past when you were successful with exercise, and about the qualities that were helpful in succeeding
9	*Finding new routes*: assess walking environment while trying a new local walking route	*Using personal strengths*: identify a “signature strength” and use it to help promote physical activity this week
10	*Planning for the future*: review progress, set goals for future increase or maintenance of physical activity	*Planning for the future*: choose 2 positive psychology skills that were most helpful, make plan to integrate moving forward

^a^Participants are asked to identify positive emotions during exercise every week throughout the intervention.

^b^SMART: specific, measurable, attainable, relevant, and time-based.

### Intervention Delivery and Fidelity

Interventionists are doctoral level psychologists or psychology doctoral students. All sessions are audio-recorded, and at least 25% of calls are reviewed for fidelity by the principal investigator using a fidelity scale developed for the trial to ensure consistency in intervention delivery. The scale measures mention of session-specific topics and procedures (eg, review of the prior week’s physical activity), use of motivational interviewing techniques, and that other psychological techniques are not used. Cases are reviewed and discussed in weekly supervision.

### Control Content

Participants randomized to the control condition receive a Fitbit and instructions for its use at their randomization visit. They are also provided with educational information about physical activity and its benefits at 4 time points throughout the intervention period (in person at randomization visit, mailed or emailed at weeks 3, 6, and 9). The study research assistant calls control participants at the midpoint of the intervention period to ensure they are receiving educational materials. These include publicly available infographics from the Centers for Disease Control and Prevention and psychoeducation material used in primary care offices affiliated with this hospital discussing overcoming barriers to physical activity and giving instruction for simple strength exercises to be completed at home. Specific physical activity goals are not provided for control participants. Fitbits are provided to all participants to ensure that group differences are not simply due to Fitbit use.

### Outcome Assessments

The primary outcomes of this study are feasibility and acceptability. Feasibility of the intervention is measured as the number of sessions attended by each participant. The intervention will be considered feasible if at least 7 of the 10 sessions are completed, on average. Intervention acceptability is measured using participant ratings on ease and utility of each intervention topic on a scale from 0 to 10 (0=“not at all easy/helpful”; 10=“very easy/helpful”). The intervention will be considered acceptable if average ratings are ≥7 out of 10.

Physical activity is assessed using accelerometers (ActiGraph GT3X-BT). At least 4 days of at least 600 minutes of wear time are required for data to be considered valid, according to established recommendations [45,46]. We will calculate average MVPA in terms of minutes per day (1952 counts per minute) and light physical activity (100-1951 counts per minute) and the daily step count. Raw data are analyzed using ActiLife (version 6.13.14; ActiGraph) in 60-second epochs. The International Physical Activity Questionnaire–Short Form is used to assess self-reported physical activity [42]. Self-efficacy for exercise is measured with the Self-Efficacy for Exercise Scale [47], exercise identity is measured with the Exercise Identity Scale [48], exercise enjoyment is measured with the Physical Activity Enjoyment Scale [49], and barriers to being active are measured with the Barriers to Being Active Quiz [50].

Psychological outcomes include positive affect measured using the Positive and Negative Affect Scale [51], optimism with the Life Orientation Test–Revised [52], symptoms of depression and anxiety with the Hospital Anxiety and Depression Scale [53], internalized weight bias with the Weight Bias Internalization Scale–Modified [54], and general self-efficacy with the General Self-Efficacy Scale [55].

Health-related outcomes include BMI, waist circumference (broadest hip and midpoint between last rib and iliac crest), percent body fat assessed with the RJL Systems Quantum IV Bioelectrical Impedance Analyzer, exercise capacity assessed using the 6-minute walk test [43], adherence to the MBS diet and vitamin regimen assessed with the Bariatric Surgery Self-Management Questionnaire [56], and general health status measured with the Short Form-12 [57]. All self-report measures have been validated in large samples.

Several physiological markers of cardiometabolic health are also measured at assessment points to test procedural feasibility in preparation for a future fully powered trial. These include blood pressure measured in mm Hg, blood lipids (low-density lipoprotein cholesterol, high-density lipoprotein cholesterol, total cholesterol, and triglycerides), HbA1c, and high-sensitivity c-reactive protein. We do not anticipate meaningful changes in these measures in this pilot trial; hence, we have not included them as formal outcomes. Table 2 summarizes the timing of the assessments.

**Table 2:** Timing of assessments [56].

Measure		Screening	Visit 1 (week 0)	Visit 2 (week 2)	10 weekly intervention calls	Visit 3 (week 14)	Visit 4 (week 28)
**Primary outcomes**							
	Feasibility	N/A^a^	N/A	N/A	Completion rate	N/A	N/A
	Acceptability	N/A	N/A	N/A	Weekly ratings	N/A	N/A
**Secondary outcome**							
	Objective moderate to vigorous physical activity	N/A	Actigraph given	Actigraph returned	N/A	Actigraph mailed 1 week prior	Actigraph mailed 1 week prior
**Additional outcomes**							
	Objective light physical activity	N/A	Actigraph given	Actigraph returned	N/A	Actigraph mailed 1 week prior	Actigraph mailed 1 week prior
	Objective steps per day	N/A	Actigraph given	Actigraph returned	N/A	Actigraph mailed 1 week prior	Actigraph mailed 1 week prior
	Self-reported physical activity	X^b^	X	N/A	Reported weekly from Fitbits and self-monitoring	X	X
	Self-efficacy for exercise	N/A	X	N/A	N/A	X	X
	Exercise identity	N/A	X	N/A	N/A	X	X
	Physical activity enjoyment	N/A	X	N/A	N/A	X	X
	Barriers to being active	N/A	X	N/A	N/A	X	X
	Positive affect	N/A	X	N/A	N/A	X	X
	Optimism	N/A	X	N/A	N/A	X	X
	Depression	N/A	X	N/A	N/A	X	X
	Anxiety	N/A	X	N/A	N/A	X	X
	Internalized weight bias	N/A	X	N/A	N/A	X	X
	MBS diet and vitamin adherence	N/A	X	N/A	N/A	X	X
	BMI	N/A	X	N/A	N/A	X	X
	Waist circumference	N/A	X	N/A	N/A	X	X
	Body fat percentage	N/A	X	N/A	N/A	X	X
	Exercise capacity	N/A	X	N/A	N/A	X	X
	General health status	N/A	X	N/A	N/A	X	X

^a^N/A: not applicable.

^b^X: measure assessed at this timepoint.

### Analytical Approach

#### Power and Sample Size

This feasibility study is not designed to detect significant between-group differences in physical activity and other outcomes; rather, its primary aim is to estimate feasibility and acceptability of the intervention. With 29 subjects receiving the intervention, we will be able to estimate the proportion who complete the intervention (feasibility) with a CI width of approximately ±0.2. We will examine the effect sizes of the intervention outcomes in addition to *P* values owing to low power.

#### Statistical Analysis Plan

We will calculate the average proportion of sessions completed to measure feasibility. The study will be considered feasible if at least 7 of 10 sessions are completed on average. Acceptability will be measured with means and SDs of participants’ ratings of session ease and utility, compared to our hypothesized target of ≥7 out of 10 for each rating. For physical activity and other psychological, behavioral, and physiological outcomes, we will model changes in each outcome using a repeated measures regression model with a fixed effect of treatment condition, a categorical effect of time, and a time by treatment interaction. The interaction will estimate the difference in the change with time comparing the treatment groups. To account for the repeated measures on each participant, we will use an unstructured covariance matrix. In addition to tests of statistical significance, which will be exploratory, given the sample size, we will calculate effect sizes to estimate the magnitude of effect of the intervention. The effect size will be estimated as the difference in the mean change with time between the groups from the interaction term divided by the estimated SD of the change with time from the unstructured covariance matrix. All tests will be considered significant based on a 2-tailed α level of .05.

## Results

Funding for this multiphase project was awarded in July 2020. The first 2 years of the award were developmental. Approval from the institutional review board for the proof-of-concept trial and RCT was attained in May 2021. The proof-of-concept trial was conducted from July 2021 through June 2022. Recruitment for the RCT began in July 2022, and study completion is anticipated by July 2024. The trial is registered at ClinicalTrials.gov [NCT04868032].

## Discussion

We hypothesize that the GOALS intervention will be feasible and acceptable and will improve physical activity and psychological well-being. After bariatric surgery, patients typically do not meet physical activity recommendations, and they receive little guidance and support to help them succeed [13]. The GOALS trial addresses this need by testing a PP-MI intervention for physical activity, which is specifically customized to the needs of this population.

While health behavior change interventions are common, the PP-MI approach is novel in its additional focus on addressing the lack of positive reinforcement that may be restraining many from developing and maintaining a consistent physical activity routine and enhancing positive psychological well-being more broadly [33]. By incorporating positive psychological skill development with motivational interviewing, self-monitoring, and goal-setting for physical activity, we hope to build participants’ self-efficacy for being active while also teaching them how to make exercise a more enjoyable experience that they will want to continue doing. Results from other versions of PP-MI interventions in other medical populations suggest that this approach is generally accepted and leads to greater well-being and MVPA, even compared to active controls [37-39]. The GOALS intervention aims to further integrate the positive psychology approach with physical activity engagement by focusing specifically on the identification and building of positive affect during physical activity and by addressing psychological barriers to being active consistently.

Another strength of the GOALS intervention is its remote delivery. In-person postoperative interventions have struggled with attendance, with common barriers including living long distances from the clinic and lack of time off from work [58-61]. By using a written manual along with weekly phone calls, participants are able to complete GOALS assignments flexibly and can more easily fit in weekly sessions from work or home. They also learn how to build physical activity into their routines in a sustainable way by finding resources in their own environments to facilitate activity rather than attending a prescribed exercise training program that has an end date.

We chose to focus the GOALS intervention on physical activity exclusively rather than also including a diet component. This was in part because patients typically receive more guidance about the postoperative diet from their surgical center than they do about physical activity. Further, we decided to focus more on the direct mental and physical health benefits of physical activity instead of encouraging exercise as a tool to lose more weight. While dietary changes are more strongly associated with weight loss than with increasing physical activity [62], patients can achieve significant health benefits from increasing physical activity independent of their weight [14-17]. By focusing on these nonweight motivators, physical activity may be more likely to improve body image [63]. When considering that long-term maintenance of physical activity after weight loss from surgery is complete, building motivators separate from weight loss is critical.

We chose the time window of 6-12 months post bariatric surgery for study enrollment based on careful consideration of several factors. By 6 months, most patients have completed standard postoperative group sessions and other care, so they may have time and interest in additional support at that time. This also allows us to target patients who have not been able to sufficiently increase physical activity on their own, as by 6 months, their physical recovery from surgery should be complete, as should their adaptation to the new diet. We limited the maximum time since surgery to 12 months to identify people who still have high motivation to make weight-related behavioral changes following their surgery.

Strengths of the study include an iteratively developed intervention incorporating patient preferences and feedback, remote delivery, randomized design, and objective measurement of physical activity and biometric outcomes. Study limitations include a small sample size with insufficient power to detect significant effects at this pilot stage and single-site delivery, which may reduce the generalizability of our results.

If the GOALS pilot RCT is feasible, acceptable to patients, and leads to improvements in physical activity and psychological outcomes, the next step will be to test the efficacy of GOALS on physical activity in a full-scale trial. Ultimately, a program such as GOALS could be integrated into clinical postoperative care as a remotely delivered, longer-term approach to promote physical activity and psychological well-being after bariatric surgery.

## Abbreviations

GOALS: Gaining Optimism After weight Loss Surgery
MVPA: moderate to vigorous physical activity
ORBIT: Obesity-Related Behavioral Intervention Trials
PP-MI: positive psychology–motivational interviewing
RCT: randomized controlled trial

## Data Availability

The data sets generated during and analyzed in this study will be available from the corresponding author on reasonable request at study completion.
